# Evaluation of *in Vivo* Antioxidant and Immunity Enhancing Activities of Sodium Aescinate Injection Liquid

**DOI:** 10.3390/molecules170910267

**Published:** 2012-08-27

**Authors:** Yong-Kun Wang, Jiang Han, Wu-Jun Xiong, Qiong-Ying Yuan, Yan-Ping Gu, Jun Li, Zhe Zhu, Hui Zhang, Cong-Jun Wang

**Affiliations:** 1 Department of Hepatobiliary & Pancreatic Diseases, School of Medicine, Shanghai East Hospital, Tongji University, Shanghai 200120, China; 2 Department of General Surgery, Pudong New Area Zhoupu Hospital, Shanghai 201318, China

**Keywords:** sodium aescinate, antioxidant, immunity, IL-1β, SOD, CAT

## Abstract

Oxidative stress is involved in the development and progression of disease. Because sodium aescinate has been reported to have immunity enhancing and antioxidative effects, we investigated its activity by employing a hepatocellular carcinoma (HCC) mouse model. Sixty BALB/c mice were randomly divided into four groups, including a 1.4 mg/kg treated group (n = 15), a 2.8 mg/kg treated group (n = 15), an untreated hepatocellular carcinoma control group (n = 15) and a normal control group (n = 15). After H22 cells were cultured for one week, we collected 2 × 10^6^ cells and injected them subcutaneously as 0.2 mL cell suspensions in sterile saline into the right shoulder region of every mouse. The animals were monitored for changes in activity, physical condition and body weight during the experiment. The next day after injection of H22 cells, animals in these test groups received one intraperitoneal injection of drug or physiological saline for 13 days. Results showed that in the sodium aescinate injection liquid (SAIL)-treated HCC mice, serum interleukin-1 beta (IL-1β), interleukin-6 (IL-6), tumor necrosis factor-alpha (TNF-α), interferon-gamma (IFN-γ), Gamma-glutamyltransferase (γ-GT), alanine transaminase (ALT), aspartate transaminase (AST) and alkaline phosphatase (ALP) levels were significantly decreased compared with normal control mice. In addition, treatment with sodium aescinate injection liquid significantly decreased blood and liver malondialdehyde (MDA) levels, increased glutathione (GSH) levels, and antioxidant enzyme [superoxide dismutase (SOD), catalase (CAT) and glutathione peroxidase (GSH-Px)] activities in a dose-dependent manner. We conclude that sodium aescinate injection liquid can decrease oxidative injury and enhance immunity functions in HCC mice.

## 1. Introduction

Reactive oxygen species (ROS) such as superoxide radicals, hydrogen peroxide (H_2_O_2_) and hydroxyl radicals are constantly generated during normal metabolism in all aerobic cells [1]. ROS have been shown to mediate many of the pathophysiological events that lead to the development of several diseases, including cancer, allergy, atherosclerosis, rheumatoid arthritis and Alzheimer’s disease [2]. To detoxify ROS, mammalian cells have evolved a complex antioxidant system that includes antioxidant enzymes (Cu/Zn-superoxide dismutase; SOD1, Mn superoxide dismutase; SOD2, catalase; CAT, glutathione peroxidase; GPx1 and glutathione reductase; GR) as well as non-enzymatic antioxidant molecules (reduced glutathione, vitamin C, vitamin E, carotenoids, *etc*.). Non-enzymatic components include glutathione (GSH), Se and some vitamins such as vitamin C and vitamin E; the antioxidant enzymes include superoxide dismutase (SOD) and glutathione peroxidase (GSH-Px), which are the major antioxidant enzymes capable of minimizing oxidative stress in the organelle [3]. The degree of lipid peroxidation is often used as an indicator of ROS-mediated damages [4] and the concentrations of malondialdehyde (MDA) in blood and tissues are generally used as biomarkers of radical-induced damage and the endogenous lipid peroxidation [5,6].

Hepatocellular carcinoma is one of the most common malignant tumors in the World. Although advances have been made in its detection and treatment, prognoses have not improved yet because of the aggressive nature of such cancers, resistance to existing chemotherapeutic agents, and lack of specific symptoms [7]. Sodium aescinate (SA) is a triterpene saponin derived from horse chestnut (*Aesculus hippocastanum*) seeds. A number of reports have indicated the therapeutic properties of the horse chestnut, such as relieving tissue oedema, recovering vasopermeability, and eliminating pressure caused by oedema [8]. SA, a natural herbal, white powder or crystalline powder, is the major active principal in extracts of the horse chestnut. SA is used as a dietary supplement and a skin care product, as well as a pharmaceutical. There are different formulations of SA in clinical applications, such as oral tablets, injections, and topical gel. Nowadays SA is widely used in the clinic for the treatment of haemorrhoids, chronic venous insufficiency [9], and encephaledema or tumefaction caused by trauma or operation [10]. In this study, we aimed to evaluate the role of sodium aescinate injection liquid on immunity and oxidative stress in HCC mice.

## 2. Results and Discussion

In the present study, we investigated the influence of sodium aescinate injection liquid on the oxidative injury and immunity status in the blood and liver of mice. In regards to tumour inhibition, it can be seen that the group treated with sodium aescinate injection liquid presented a 20.07 and 31.83% inhibition as compared with the untreated model control group (Table 1).

**Table 1:** Sodium aescinate injection liquid affecting tumour inhibition rate.

Group	Tumour weight (g)	Inhibition rate (%)
I	-	-
II	1.47 ± 0.13	-
III	1.18 ± 0.12 ^d^	20.07
IV	1.01 ± 0.09 ^d^	31.83

^d^ *p* < 0.01, compared with group II. I: normal control group; II: untreated model control (HCC) group; III: HCC + sodium aescinate injection liquid (1.4 mg/kg b.w.) group; IV: HCC + sodium aescinate injection liquid (2.8 mg/kg b.w.) group.

Several inflammatory cytokines, particularly TNF-α, IL-1β and IL-6, are known to play key roles in the induction and perpetuation of inflammation in macrophages. These cytokines play pivotal roles in the induction of the innate immune response, as well as in the determination of the magnitude and nature (Th1 *vs*. Th2) of the acquired immune response [11]. IFN-α, which is induced by T cells, NK cells, and macrophages, inhibits the growth of cancer cells and promotes type 1 immune responses and differentiation of cytotoxic NK and T cells [12]. Tumor necrosis factor-α (TNF-α) is a potent anti-tumor cytokine that enhances the activity of macrophages, NK cells, and cytotoxic T-cells [13,14]. Table 2 shows that the serum IL-1β, IL-6, TNF-α, and IFN-γ levels were significantly higher in the untreated model control group than in the normal group (*p* < 0.01). This result indicates that H22 cells treatment stimulated secretion of proinflammatory cytokines (IL-1β, TNF-α, IFN-γ and IL-6) in mouse peritoneal macrophages. The low dose of sodium aescinate injection liquid dose-dependently markedly reduced the serum IL-1β, IL-6, TNF-α, and IFN-γ levels in the SAIL groups compared to the untreated model control group (*p* < 0.05; *p* < 0.01). In many tumour diseases, serum TNF-α and IFN-gamma levels are increased, its expression upregulation is close relationship to tumour theraphy. In present study, serum TNF-α and IFN-gamma levels are increased in HCC mice. However, after SAIL treatment, serum NF-α and IFN-gamma levels are decreased in HCC mice. We suppose that SAIL play its antitumour activities by inducing cell apoptosis *etc*. As a result, this inhibit TNF-α and IFN-gamma *etc*. into blood.

**Table 2:** Sodium aescinate injection liquid affecting serum IL-1β, IL-6, TNF-α, and IFN-γ levels.

Group	IL-1β (pg/mL)	IL-6 (pg/mL)	TNF-α (pg/mL)	IFN-γ (pg/mL)
I	115.28 ± 9.49	209.43 ± 19.57	3.76 ± 0.33	32.38 ± 2.48
II	154.03 ± 13.18 ^b^	286.27 ± 24.08 ^b^	7.03 ± 0.49 ^b^	41.27 ± 3.88 ^b^
III	133.51 ± 10.39 ^d^	241.96 ± 22.15 ^d^	5.29 ± 0.41 ^d^	38.46 ± 3.45 ^c^
IV	118.19 ± 9.99 ^d^	215.27 ± 17.83 ^d^	4.17 ± 0.32 ^d^	34.93 ± 2.67 ^d^

^b^ *p* < 0.01, compared with group I; ^c^ *p* < 0.05, ^d^ *p* < 0.01, compared with group II. I: normal control group; II: untreated model control (HCC) group; III: HCC + sodium aescinate injection liquid (1.4 mg/kg b.w.) group; IV: HCC + sodium aescinate injection liquid (2.8 mg/kg b.w.) group.

Transaminases (AST and ALT), ALP, γ-GT and LDH activity exhibited a general increase in H22-treated mice. Table 3 shows that the serum γ-GT, ALT, AST and ALP levels were significantly higher in the untreated model control group than in the normal group (*p* < 0.01). The observed elevation in the activity of these enzymes in untreated model control group is in agreement with the previous studies of who found that the activity of AST, ALT and ALP was increased significantly following H22 cells treatment to mice. The liver enzymes are normally found in circulation in small amounts because of hepatic growth and repair. As a liver specific enzyme ALT only significantly elevated in hepatobiliary disease, increase in AST level, however, can occur in connection with damages of heart or skeletal muscle as well as of liver parenchyma [15]. Consequently, elevated activity of ALT and AST observed in the current study could be a common sign of impaired liver function. Also, an elevation of both γ-GT and LDH enzymes activity was recorded. Sodium aescinate injection liquid dose-dependently markedly reduced the serum γ-GT, ALT, AST and ALP levels in the SAIL groups compared to the untreated model control group (*p* < 0.05; *p* < 0.01). Our present work indicated that Sodium aescinate injection liquid is useful for repair of liver function.

**Table 3:** Sodium aescinate injection liquid affecting serum γ-GT, ALT, AST and ALP levels.

Group	γ-GT (U/100 mL)	ALT (U/L)	AST (U/L)	ALP (U/L)
I	2.74 ± 0.18	60.32 ± 5.39	94.81 ± 7.82	80.35 ± 5.38
II	10.75 ± 1.22 ^c^	102.69 ± 8.49 ^b^	140.27 ± 10.58 ^b^	139.28 ± 11.29 ^b^
III	7.23 ± 0.57 ^d^	83.19 ± 6.02 ^d^	129.71 ± 11.03 ^c^	115.03 ± 10.01 ^c^
IV	5.38 ± 0.42 ^d^	72.17 ± 6.11 ^d^	114.38 ± 9.37 ^d^	97.36 ± 6.82 ^d^

^b^ *p* < 0.01, compared with group I; ^c^ *p* < 0.05, ^d^ *p* < 0.01, compared with group II. I: normal control group; II: untreated model control (HCC) group; III: HCC + sodium aescinate injection liquid (1.4 mg/kg b.w.) group; IV: HCC + sodium aescinate injection liquid (2.8 mg/kg b.w.) group.

Free radicals and reactive oxygen species (ROS) are continuously produced in the human body. These oxygen species are the cause of cell damage and the progression of tumour cells to cancer cells. Therefore, tissues must be protected from oxidative injury through intracellular (SOD, GSH-Px and catalase) as well as extracellular (vitamins, micronutrients, antioxidants originated from herbs) antioxidants [16].

MDA levels have been used as a marker for lipid peroxidation in cancer [17,18,19]. The high levels of MDA in cancerous conditions could result from the deterioration of antioxidant defenses, as studied by Szatrwoski and Nathan [20]. The current biochemical alterations coincided with the present marked increase of blood and hepatic MDA, so the negative results associated with toxic nitrosamine compounds may be attributed to the hepatic injury induced by superoxide anions and hydroxyl radicals which cause oxidative damage to cell membrane resulted in an increase in the activity of liver enzymes [21].

As a biomarker for lipid peroxidation, blood and hepatic lipid peroxidation was determined by measuring the TBARS concentration in mice. H22 cells treatment evoked an increase in the concentration of blood and hepatic MDA, indicating oxidative damage to cell lipids. The significant increase in hepatic lipid peroxides of mice, explains observed leakage of cellular ALT, AST and ALP to circulation due to liver injury. TBARS concentration in blood and liver of SAIL-treated mice were significantly decreased in a dose-dependent manner compared to untreated model control mice (Table 4). 

SOD, CAT and GSH-Px enzymes are important scavengers of superoxide ion and hydrogen peroxide. These enzymes prevent generation of hydroxyl radical and protect the cellular constituents from oxidative damage [22]. Superoxide dismutase (SOD) is an enzyme extensively used as a biochemical indicator of pathological states associated with oxidative stress [23]. It is the only enzymes dismounting superoxide radicals. CAT converts H_2_O_2_ to H_2_O and GPx catalyses the transformation of H_2_O_2_ into harmless byproducts. During H_2_O_2_ scavenging, GSH is oxidized to GSSG by GPx. The decrease in GSH levels, GSH/GSSG ratio and GSH-Px and CAT activities and increase in GSSG hepatic content indicates the severity of the oxidative stress-induced by liver cancer. Decreases in GSH levels, as well as in activities of CAT and GSH-Px, have been reported in hepatic injury induced by toxicants [24,25,26]. It has been demonstrated that different natural compounds and polyphenols induce GSH as one of the principal anticarcinogenic mechanisms [27,28,29].

**Table 4:** Sodium aescinate injection liquid affecting serum and liver MDA, GSH, SOD, CAT and GSH-Px levels.

Group		MDA	GSH	SOD	CAT	GSH-Px
I	serum	4.28 ± 0.27	173.14 ± 12.84	261.33 ± 18.04	48.03 ± 2.81	55.28 ± 4.44
	liver	3.37 ± 0.27	152.85 ± 13.65	216.42 ± 18.48	49.02 ± 3.01	41.39 ± 3.33
II	serum	8.57 ± 0.71 ^b^	90.21 ± 7.03 ^b^	150.28 ± 12.25 ^b^	22.16 ± 1.94 ^b^	33.21 ± 2.73 ^b^
	liver	8.02 ± 0.71 ^b^	98.27 ± 8.02 ^b^	163.15 ± 13.29 ^b^	21.47 ± 1.69 ^b^	23.07 ± 2.01 ^b^
III	serum	6.44 ± 0.54 ^d^	136.21 ± 10.37 ^d^	197.24 ± 14.81 ^d^	35.19 ± 2.69 ^d^	42.09 ± 2.88 ^d^
	liver	6.81 ± 0.53 ^d^	130.22 ± 11.26 ^d^	188.22 ± 14.06 ^d^	31.77 ± 1.52 ^d^	30.82 ± 2.31 ^d^
IV	serum	5.25 ± 0.43 ^d^	155.73 ± 13.22 ^d^	257.13 ± 22.04 ^d^	42.83 ± 3.01 ^d^	51.37 ± 3.53 ^d^
	liver	4.62 ± 0.35 ^d^	148.91 ± 12.53 ^d^	204.15 ± 17.39 ^d^	43.05 ± 3.57 ^d^	38.04 ± 2.86 ^d^

^b^ *p* < 0.01, compared with group I; ^d^ *p* < 0.01, compared with group II. I: normal control group; II: untreated model control (HCC) group; III: HCC + sodium aescinate injection liquid (1.4 mg/kg b.w.) group; IV: HCC + sodium aescinate injection liquid (2.8 mg/kg b.w.) group.

The activities of the three blood and hepatic antioxidant enzymes CAT, SOD, and GPx and GSH were evaluated in the mice. As shown in Table 4, the blood and hepatic SOD, CAT and GPx activities and GSH level were significantly lower in the untreated model control group than in the normal control group (*p* < 0.05). Sodium aescinate injection liquid dose-dependently markedly enhanced the blood and hepatic SOD, CAT and GPx activities and GSH level in SAIL-treated mice compared to untreated model control mice. The decrease in the activities of these (GSH-Px, CAT, SOD) enzymes result in the involvement of deleterious oxidative changes and also insufficient availability of GSH. The result of the SOD, CAT and GSH-Px activities clearly shows that Sodium aescinate injection liquid contains a free radical scavenging activity, which could exert a beneficial action against pathological alteration caused by the presence of O_2_• and OH•. This action could involve mechanism related to scavenging activity.

In light microscopic examinations, histopathological changes were observed in the livers of all experiment groups compared with those of controls. In the normal control mice, normal liver histologic aspect with central vein and radiating hepatic cords was seen. HCC mice exhibited severe histopathological changes, such as mononuclear cells infiltration in the parenchymatous tissue and portal area, congestion, enlargement of the hepatic sinusoids and enlargement of the central and the portal veins and hepatocellular damage. In contrast, the histological examination of tissue sections from mice treated with sodium aescinate injection liquid showed an improvement of liver morphology, aside from mild inflammation. Necrotic cells and vacuolization are nearly absent. 

## 3. Experimental

### 3.1. Chemicals

Sodium aescinate injection liquid was purchased from Shandong LuYe Pharmaceutical Company Limited (Yantai, China).

### 3.2. Cell Culture

H22 cells were cultured in RPMI 1640 medium buffer, supplemented with 10% heat-inactivated fetal calf serum (FCS), 100 U/mL penicillin and 100 μg/mL streptomycin. Cells were incubated at 37 °C in a humidified atmosphere containing 5% carbon dioxide (CO_2_) for experiments. The medium buffer was exchanged every day.

### 3.3. Transplantation of H22 Cells as Solid Tumors in Nude Mice and Treatment of Animals

Human cancer xenograft models were established using the methods reported previously. Sixty BALB/c mice were randomly divided into four groups, including 1.4 mg/kg treated group (III) (n = 15), 2.8 mg/kg treated group (IV) (n = 15), untreated hepatocellular carcinoma control group (II) (n = 15) and normal control group (I) (n = 15). After H22 cells were cultured for one week, 2 × 10^6^ cells were collected and injected subcutaneously as 0.2 mL cell suspensions in sterile saline into the right shoulder region of every mouse. The animals were monitored for changes in activity, physical condition and body weight during the experiment. The next day after injection of H22 cells, animals in these test groups received one intraperitoneal injection of drug or physiological saline for 13 days. The mice were sacrificed by cervical dislocation on the next day after stopping injection. Tumors were dissected, weighed individually. The tumor inhibition rate was calculated using the following formula: tumor inhibitory rate = (tumor weight of negative control group − tumor weight of tested group)/tumor weight of negative control group ×100.

### 3.4. Biochemical Assay

IL-1β, IL-6, TNF-α and IFN-γ levels were measured with ELISA kits (ShenZhen MeiJing Bioengineering Ltd., ShenZhen, China). The serum activity of ALT, AST and γ-GT was determined using a commercial kit in accordance with manufacturers’ instructions (ELITech Group, Paris, France). The absorbance was quantified in a microplate reader ELx800 BioTek (Winooski, VT, USA).

Lipid peroxidation was estimated by measuring thiobarbituric acid-reactive substances (TBARS) and expressed in terms of malondialdehyde (MDA) content, according to the method of Draper and Hadley [30]. The MDA values were calculated using 1,1,3,3-tetraethoxypropane as standard and expressed as nmol of MDA/g.

Glutathione (GSH) was measured following the method of Fukuzawa and Tokumura [31]. Supernatant (200 μL) was added to 0.25 M sodium phosphate buffer (1.1 mL, pH 7.4) followed by the addition of DTNB 0.04% (130 μL). Finally, the mixture was brought to a final volume of 1.5 mL with distilled water and absorbance was read in a spectrophotometer at 412 nm and results were expressed as μg GSH/μg protein.

The activity of superoxide dismutase (SOD) was assayed by monitoring its ability to inhibit the photochemical reduction of nitroblue tetrazolium (NBT). Each reaction mixture (1.5 mL) contained 100 mM Tris/HCl (pH 7.8), 75 mM NBT, 2 μM riboflavin, 6 mM EDTA, and 200 μL of supernatant. Monitoring the increase in absorbance at 560 nm followed the production of blue formazan. One unit of SOD is defined as the quantity required to inhibit the rate of NBT reduction by 50% as described by Winterbourn *et al*. [32].

The catalase (CAT) activity was determined according to the Aebi method [33]. The rate of H_2_O_2_ decomposition was followed by monitoring absorption at 240 nm. One unit of CAT activity is defined as the amount of enzymes required to decompose 1 μmol of hydrogen peroxide in 1 min. 

Glutathione peroxidase (GSH-Px) activity in tissue was assessed by the method of Paglia and Valentine [34]. One unit of GSH-Px was defined as the oxidation by H_2_O_2_ of 1 μmol of reduced glutathione to oxidized glutathione per min at pH 7 at 25 °C.

### 3.5. Histopathological Examination

Liver specimens were fixed in 4% formalin and then embedded in paraffin. Tissue sections (4 μm-thick) were stained with hematoxylin-eosin (HE) for routine examination.

### 3.6. Statistical Analysis

All results are expressed as group mean ± S.D. Comparison with appropriate controls was performed using one-way ANOVA (p-values of less than 0.05 were regarded as significant). Significant values were assessed with Duncan’s multiple rang test. Data was analyzed using the statistical package “SPSS 12.0 for Windows”.

## 4. Conclusions

Sodium aescinate injection liquid may contain bioactive components with antioxidant activity in scavenging free radicals such as superoxide anions and H_2_O_2_ as well as decreasing the MDA level for the reduction of lipid peroxidation and with immunity enhancing activity in HCC mice. 
